# The complete chloroplast genome of *Psychotria asiatica*, Linnaeus 1759 (Rubiaceae)

**DOI:** 10.1080/23802359.2022.2077669

**Published:** 2022-05-26

**Authors:** Xiaoshan Geng, Rong Chen, Bo Li, Yulin Zhu, Qin Liu

**Affiliations:** Guangxi Key Laboratory of Agricultural Resources Chemistry and Biotechnology, Yulin Normal University, Yulin, China

**Keywords:** *Psychotria asiatica*, chloroplast genome, phylogenetic analysis

## Abstract

*Psychotria asiatica* is a typical traditional medicinal plant. Herein, we acquired and characterized the complete chloroplast (cp) genome sequence of *P. asiatica* to provide genomic resources for conservation genetics and phylogenetic studies of *P. asiatica*. The cp genome of *P. asiatica* is 154,652 bp in length and consists of a large single-copy (LSC) region with 85,106 bp, a small single-copy (SSC) region with 17,960 bp, and two inverted repeat regions (IRs) with 25,793 bp. The cp genome of *P. asiatica* comprises 127 genes, including 82 protein-coding genes, 37 tRNA genes, and eight rRNA genes. The phylogenetic result confirmed that *P. asiatica* was closely related to *Psychotria kirkii* within the Rubiaceae.

*Psychotria asiatica* Linnaeus (1759), a synonym of *Psychotria rubra*, is a small evergreen shrub in the Rubiaceae family that grows widely in tropical and subtropical regions of Asia. Typically, it grows in warm and humid environments at altitudes of 20–1500 m. The leaves are 8–20 cm long and 2.5–7 cm wide, mostly with blades that are oblong, elliptic-oblong, or inverted lanceolate oblong. Its inflorescences are cymose to paniculiform and its cyme is often terminal, with a very short peduncle, with three forks near the nodes. The roots, stems, and leaves of *P. asiatica* are used in traditional Chinese medicine to treat various diseases such as colds, diphtheria, dysentery, and injuries (Tang et al. 2018). Previous studies on *P. asiatica* have mainly focused on its chemical composition and pharmacological effects. In contrast, the molecular biology and phylogeny of *P. asiatica* have received little attention. To date, only 43 nucleotide sequences have been released in the NCBI, except for the chloroplast (cp) genome in this study. Thus, the cp genome sequence of *P. asiatica* is reported and characterized here to provide genomic resources for conservation genetics and phylogenetic studies of *P. asiatica*.

Fresh leaves of *P. asiatica* were collected from Yulin Normal University (110.184°E, 22.666°N) in Yulin, Guangxi, China. The voucher specimen (JJ-YNU-001) was deposited in the Herbarium of Yulin Normal University (https://syy.ylu.cn/index.html, Yulin Zhu, gxzyl@163.com). Total genomic DNA was extracted by the CTAB method and sequenced by the Hiseq 4000 platform (Illumina, San Diego, CA) with 150 bp paired-end (PE) sequencing. The adapters and low-quality reads were trimmed from the raw reads using Trimmomatic (Bolger et al. 2014) with the following settings: ILLUMINACLIP 2:30:10, LEADING: 3, TRAILING: 3 SLIDINGWINDOW: 4:25, and MINLEN: 100. The clean reads were de novo assembled and annotated by the GetOrganelle toolkit (Jin et al. 2020) and CPGAVAS2 (Shi et al. 2019), respectively, using the default parameters. The cp sequence of *P. asiatica* was submitted to NCBI under the accession number of MZ958829.

The cp genome of *P. asiatica* is 154,652 bp in length and consists of a large single-copy (LSC with 85,106) region, a small single-copy (SSC with 17,960 bp) region, and two inverted repeat regions (IRs with 25,793 bp). The overall GC content of the *P. asiatica* cp genome is 37.68% and the GC content of the LSC, SSC, and IR regions is 35.49%, 31.49%, and 43.40%, respectively. The cp genome of *P. asiatica* contains 127 genes, including 82 protein-coding genes, 37 tRNA genes, and eight rRNA genes. Among those genes, six tRNA genes and 12 protein-coding genes contain introns. Three genes, *ycf3*, *clpP*, and *rps12*, have two introns, while the remaining 15 genes have only one intron.

To identify the phylogenetic position of *P. asiatica*, a maximum-likelihood (ML) tree was reconstructed based on the cp genome sequences of 13 Rubiaceae species and one outgroup from the Acanthaceae. Multiple sequence alignment of cp genomic sequences was performed using MAFFT (Rozewicki et al. 2019) with default parameters, and the ML tree was reconstructed using the IQ-TREE (Nguyen et al. 2015) with 1000 bootstrap replications and GTR + F+I + G4 model (Figure 1). *Alstonia scholaris*, which belongs to the Acanthaceae, was used as an outgroup. The phylogenetic result showed that *P. asiatica* was closely related to *Psychotria kirkii* within the Rubiaceae, with bootstrap support values of 100%. The complete cp genome of *P. asiatica* could provide genomic data for further genetic and evolutionary studies on Rubiaceae species.

**Figure 1.**
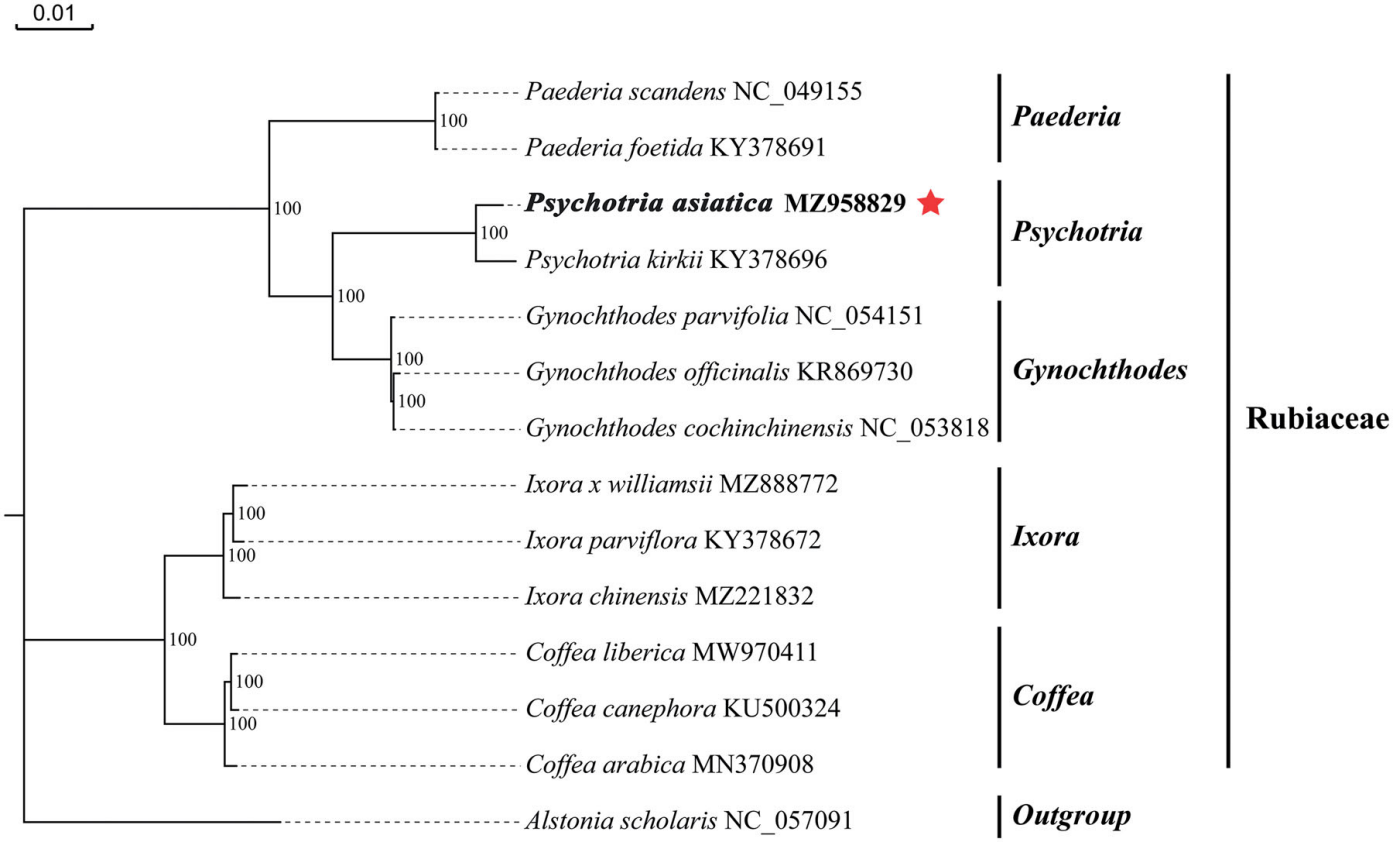
The maximum-likelihood (ML) phylogenetic tree is based on cp genome of *P. asiatica* and other 13 species. Bootstrap values are based on 1000 replicates. The numbers on branches are bootstrap support values.

## Ethical approval

The data collection of plants was carried out with the permission of Yulin Normal University and complied with local (Yulin, Guangxi, China) legislation.

## Author contributions

Qin Liu and Rong Chen were involved in the conception and design; Xiaoshan Geng and Bo Li contributed the sample collection; Xiaoshan Geng and Qin Liu performed the analysis and interpretation of the data; Yulin Zhu and Rong Chen contributed the drafting of the paper; Qin Liu and Yulin Zhu revised it critically for intellectual content. All authors were involved in the final approval of the version to be published. All authors agree to be accountable for all aspects of the work.

## Data Availability

The genome sequence data that support the findings of this study are openly available in GenBank of NCBI at https://www.ncbi.nlm.nih.gov under the accession no. MZ958829. The associated BioProject, SRA, and Bio-Sample numbers are PRJNA812717, SRR18218093, and SAMN26424688, respectively.
